# An evaluation of a state-level fruit and vegetable incentive’s impacts on food and nutrition security among SNAP participants

**DOI:** 10.3389/fnut.2025.1706200

**Published:** 2025-11-26

**Authors:** Vanessa M. Oddo, Julien Leider, Alison Tovar, Maya K. Vadiveloo, Emily Elenio, Lisa M. Powell

**Affiliations:** 1 Department of Kinesiology and Nutrition, College of Applied Health Sciences, University of Illinois Chicago, Chicago, IL, United States; 2 Institute for Health Research and Policy, School of Public Health, University of Illinois Chicago, Chicago, IL, United States; 3 Department of Behavioral and Social Sciences, School of Public Health, Brown University, Providence, RI, United States; 4 Department of Nutrition, College of Health Sciences, The University of Rhode Island, Kingston, RI, United States; 5 Division of Health Policy and Administration, School of Public Health, University of Illinois Chicago, Chicago, IL, United States

**Keywords:** nutrition policy, quasi-experimental design, fruits and vegetables, food insecurity, nutrition insecurity

## Abstract

**Objective:**

To test the short-term effect of a state-level fruit and vegetable incentive (FVI) on food and nutrition security among Supplemental Nutrition Assistance Program (SNAP) participants.

**Methods:**

In January 2024, Rhode Island (RI) implemented a FVI, which provided a $0.50 credit for every $1.00 spent on fresh FV up to $25/month. Using a difference-in-differences approach, we used separate adjusted logistic regression models to evaluate FVI impacts on household food and nutrition insecurity and perceived FV affordability, in RI relative to changes in the comparison site of Connecticut (CT), ~6 months after implementation.

**Results:**

At baseline, participants’ (*N* = 725) mean age was ~35 years old, ~95% were women, ~40% identified as Hispanic, and ~25% perceived that they could not afford FV. Food (55–59%) and nutrition (30–34%) insecurity were high in both states. After implementation, neither food [Odds Ratio (OR): 1.14; 95% Confidence Interval (CI): 0.84–1.55] nor nutrition insecurity (OR: 0.92; 95% CI: 0.63–1.34) changed in RI relative to CT. There were also no changes over time in perceived FV affordability in RI versus CT (OR: 0.80; 95% CI: 0.54–1.18).

**Conclusion:**

We did not observe short-term changes in food or nutrition insecurity associated with the FVI provided to SNAP participants. Future analyses should evaluate longer-term policy effects.

## Introduction

Food and nutrition insecurity remain critical public health issues in the United States (U.S.). In 2023, 13.5% of U.S. households experienced food insecurity, commonly defined as lacking access to *enough* food for a healthy life (1). A related construct, nutrition security, emphasizes the ability to access and afford food for a *nutritionally adequate* diet that promotes health and prevents disease (2, 3). Using a proxy measure, prior evidence suggests ~36% of Americans are nutrition insecure (4), with a higher prevalence (~50%) previously observed among Supplemental Nutrition Assistance Program (SNAP) participants (5). Both food and nutrition insecurity can negatively impact diet quality (6, 7), which in turn, contributes to diet-related diseases among individuals with low income.

SNAP provides food benefits to individuals with low income, with the primary goal of reducing food insecurity in the U.S. While SNAP participation does decrease food insecurity among participants (8, 9), many remain food insecure (8), suggesting that additional structural- (e.g., transportation) and individual-level barriers persist, including affordability, particularly for nutrient-dense foods (10). Nutrition incentive programs are one emerging strategy that aim to decrease food and nutrition insecurity by reducing financial burden among individuals with low income in the U.S.

Prior evaluations suggest that fruit and vegetable incentive (FVI) programs have reduced food insecurity among individuals with low income (11–13). Despite this prior evidence, few FVI have implemented programs at scale, many require enrollment to participate, and most evaluations do not include a control group. Moreover, none have evaluated their effects on household nutrition insecurity.

In January 2024, Rhode Island (RI) implemented the *Eat Well, Be Well* (EWBW) program, the first statewide FVI program automatically delivered to all SNAP households via EBT cards. Using a difference-in-differences approach, we tested the effect of EWBW on household food and nutrition insecurity among adult SNAP participants, with Connecticut (CT) serving as the comparison state. Study results can contribute to our understanding of the extent to which FVI programs affect food and nutrition insecurity, which may help inform the future direction of food assistance programs in the U.S.

## Methods

### Study overview

RI implemented EWBW in January 2024, which provided an automatic credit to each SNAP participant’s EBT card of $0.50 for every $1.00 spent on fresh FV sold by select participating SNAP retailers (Stop & Shop, Walmart), up to $25.00/month. A total of $11.5 million was allocated to the program, which will continue until funds are exhausted. In 2024, $3.2 million in extra SNAP benefits were issued as part of EWBW. Detailed elsewhere, the secondary aim of the larger evaluation was to evaluate the effect of the FVI on diet quality, measured via the Healthy Eating Index (HEI)-2015, employing a quasi-experimental design (14, 15). CT was selected as the comparison state based on geographic proximity and demographic, socioeconomic, and political similarity. Notably, 2023 data from the American Community Survey suggest that the percent of households participating in SNAP was similar in Rhode Island (13%) and Connecticut (12%) (16). We also aimed to evaluate the effect of the FVI on household food and nutrition insecurity, which was the focus of these analyses.

### Study procedures

We recruited participants from RI and CT prior to policy implementation (May to September 2023) via in-person events, community partnerships, and text messages sent to Women, Infants, and Children (WIC) program participants. Participants that consented to participation completed a screener via Qualtrics to verify eligibility. Inclusion criteria included: speaking and reading English or Spanish; being ≥ 18 years old; currently participating in SNAP; living in RI or CT; and having access to email and a text message-capable phone. Eligible participants filled out a food frequency questionnaire (FFQ) through VioScreen and a sociodemographic survey in Qualtrics (*n* = 1,367) (15, 17).

Between June and September 2024, previously enrolled participants (*n* = 1,363) were recontacted for follow-up, and asked to verify their SNAP status by submitting a photo of a grocery receipt that showed their SNAP EBT card number, during the prior 30 days. Verified and eligible participants who responded to follow-up outreach were asked to complete a second FFQ and sociodemographic questionnaire. Participants received a $50 electronic gift card at each time period. Study procedures are detailed elsewhere (15).

### Analytic sample

Consistent with our primary analyses, of the 837 individuals that completed the follow-up survey, participants were excluded if they moved from RI to CT (*n* = 1), were missing data on covariates (*n* = 3), reported extreme fruit and vegetable intake (>7.2 cup equivalents /1,000 kcal at baseline or >8 cup equivalents/1000 kcal at follow-up, including fruit juice; *n* = 10), or reported extreme overall dietary intake [≤500 kcal or ≥5,500 kcal (*n* = 87), or ≤25 different foods from FFQ (*n* = 11)]. The final analytical sample included 725 SNAP participants in RI and CT.

### Key study variables

Our online survey queried respondents on demographic and household information, food insecurity, nutrition insecurity, food access barriers, and diet (17). For these analyses, household food and nutrition insecurity served as our primary dependent variables. Food insecurity was defined using the 6-item USDA Module (18) and a binary measure was created based on a score of 0–1 (food secure) versus 2–6 (food insecure). A binary nutrition insecurity measure was defined using the 1-item measure developed by the Center for Nutrition and Health Impact (19) based on responses in the affirmative to the question, “In the last 30 days, we worried that the food we were able to eat would hurt our health and well-being.” At follow-up only, we also collected data on the full 4-item nutrition insecurity measure with a 12-month reference period (19).

Individuals were asked to check all that apply to several perceived barriers for buying and preparing FV, including not being able to afford FV; limited transportation options; limited variety of FV sold at the store; disklike of FV among the household members; and not knowing how to prepare FV, among others. Non-financial barriers for purchasing FV likely persisted following EWBW (e.g., transportation, nutrition education). However, given that cost was the most direct mechanism through which the FVI could affect food and nutrition insecurity, we secondarily explored whether perceived FV affordability (a binary variable) was impacted by the FVI.

### Statistical analyses

We computed summary statistics by state and time period and then estimated adjusted logistic regression difference-in-differences models with robust standard errors, clustered on participant. Based on prior literature (5, 20–25) and variables hypothesized to affect food and nutrition insecurity, we controlled for the following covariates: education, employment, marital status, household size, living arrangement, race and ethnicity, gender, baseline age, and household receipt of WIC.

Consistent with our primary analysis focused on diet (14), we also conducted exploratory analyses stratified by lower (*n* = 363, 0.10–1.79 cup equivalents/1000 kcal) and upper (*n* = 362, >1.79–7.13 cup equivalents/1000 kcal) halves of the baseline FV intake distribution.

Analyses were conducted in Stata/MP 18.0. This study was approved by the institutional review boards at Brown University and University of Rhode Island.

## Results

Largely, the demographic characteristics of participants were similar by state and time period (Table 1). At baseline, participants’ mean age was ~35 years old, ~95% were women, and ~40% identified as Hispanic. About half of the sample had ≥some college, but this was higher in CT (60%) than RI (47%). At baseline, 42 and 49% of the sample was employed in RI and CT, respectively; the percent of the sample that was employed was somewhat higher in both states at follow-up (47% in RI, 53% in CT). Most participants also received WIC benefits at baseline (77% in RI, 83% in CT), but this was lower in both states at follow-up (67% in RI, 70% in CT). In both states, ~25% of the sample perceived that they could not afford FV at baseline; this was slightly lower in RI at follow-up (23%). Food (55–59%) and nutrition (30–34%) insecurity were highly prevalent at baseline in both states; both were slightly lower at follow-up, but remained around 53 and 30%, respectively.

**Table 1:** Characteristics of analytical sample of SNAP participants in Rhode Island and Connecticut^a^.

Characteristic	Rhode Island (*N* = 364)		Connecticut (*N* = 361)	
	Baseline	Follow-up	Baseline	Follow-Up
	Mean (SD) or *n* (%)	Mean (SD) or *n* (%)	Mean (SD) or *n* (%)	Mean (SD) or *n* (%)
Food and nutrition outcomes				
Food insecure	201 (55.22%)	193 (53.02%)	213 (59.00%)	193 (53.46%)
Nutrition insecure	111 (30.49%)	99 (27.20%)	123 (34.07%)	117 (32.41%)
Cannot afford fruits and vegetables	95 (26.10%)	82 (22.53%)	89 (24.65%)	91 (25.21%)
Participant/household characteristics				
Woman	342 (93.96%)	–	348 (96.40%)	–
Baseline age	35.16 (10.56)	–	33.83 (9.54)	–
Employed	153 (42.03%)	171 (46.98%)	178 (49.31%)	190 (52.63%)
Marital status				
Married or living with a partner	93 (25.55%)	97 (26.65%)	112 (31.02%)	118 (32.69%)
Never married, divorced, widowed, or separated	253 (69.51%)	250 (68.68%)	229 (63.43%)	232 (64.27%)
Prefer not to answer	18 (4.95%)	17 (4.67%)	20 (5.54%)	11 (3.05%)
Household size	3.80 (1.59)	3.88 (1.60)	3.91 (1.62)	3.90 (1.62)
Living arrangement other than housing where own or pay to stay	36 (9.89%)	24 (6.59%)	38 (10.53%)	32 (8.86%)
Race and ethnicity				
Hispanic	144 (39.56%)	–	145 (40.17%)	–
Non-Hispanic Black or African American	33 (9.07%)	–	88 (24.38%)	–
Non-Hispanic White	145 (39.84%)	–	98 (27.15%)	–
Non-Hispanic Other	42 (11.54%)	–	30 (8.31%)	–
Completed some college or more	170 (46.70%)	–	217 (60.11%)	–
Received WIC in past 3 months	279 (76.65%)	245 (67.31%)	298 (82.55%)	254 (70.36%)
Received Summer EBT for children in past 3 months^b^				
Yes	–	221 (60.71%)	–	145 (40.17%)
No	–	119 (32.69%)	–	177 (49.03%)
Not sure	–	24 (6.59%)	–	39 (10.80%)

SD, standard deviation; SNAP, Supplemental Nutrition Assistance Program; WIC, Women, Infants, and Children program.

a *N* = 364 participants in Rhode Island and 361 participants in Connecticut for whom data were obtained on all relevant covariates at both time points and who were not excluded due to extreme reported fruit and vegetable intake (>7.2 cup equivalents/1000 kcal at baseline or >8 cup equivalents/1000 kcal at follow-up, including fruit juice) or dietary intake (≤500 kcal, ≥5,500 kcal, or ≤25 different foods from the food frequency questionnaire).

b Data on receipt of Summer EBT for children were only obtained at follow-up because this program did not start in either Rhode Island or Connecticut until 2024, after baseline data collection was already complete.

Overall, ~6 months after the implementation of the FVI program, neither food [Odds Ratio (OR): 1.14; 95% Confidence Interval (CI): 0.84–1.55] nor nutrition insecurity (OR: 0.92; 95% CI: 0.63–1.34) changed in RI relative to CT (Table 2). We also did not observe changes in food nor nutrition insecurity in RI, compared to changes in CT, when results were stratified by baseline FV intake. Nor did we observe changes over time in perceived affordability of FV in RI versus CT (OR: 0.80; 95% CI: 0.54–1.18).

**Table 2:** Results of difference-in-differences regression models examining changes in food and nutrition insecurity and reported barriers to buying and preparing fruits and vegetables in Rhode Island relative to Connecticut^a^.

Outcome	Overall	Lower FV intake^b^	Higher FV intake^b^
	OR (95% CI)	OR (95% CI)	OR (95% CI)
Food insecurity	1.14 (0.84–1.55)	1.18 (0.75–1.85)	1.11 (0.71–1.73)
Nutrition insecurity	0.92 (0.63–1.34)	1.24 (0.73–2.09)	0.65 (0.37–1.15)
Cannot afford FV	0.80 (0.54–1.18)	0.68 (0.38–1.21)	0.92 (0.55–1.56)

CI, confidence interval; FV, fruit and vegetables; OR, odds ratio.

a *N* = 725 Rhode Island and Connecticut SNAP participants. Difference-in-differences results shown from logistic regression models with robust standard errors clustered on participant adjusting for household receipt of WIC in the past 3 months (no or not sure; yes), education ( ≤ high school; ≥ some college), employment (employed; not employed), marital status (married or living with a partner; never married, divorced, widowed, or separated; prefer not to answer), household size (continuous), living arrangement [housing where own or pay to stay; other arrangement (e.g., shelter, car)], race and ethnicity (Hispanic; Non-Hispanic Black; Non-Hispanic White; Non-Hispanic Other), gender (woman; nonbinary or man), and baseline age (continuous).

b Lower FV intake (363 participants): 0.10–1.79 cup equivalents/1,000 kcal; higher FV intake (362 participants): >1.79–7.13 cup equivalents/1,000 kcal.

## Discussion

To our knowledge, this study is the first to investigate a statewide automatic SNAP FVI in relation to food and nutrition security, using a quasi-experimental design. All difference-in-differences regression results were null.

Our findings contrast with a recent systematic review reporting lower household food insecurity among individuals with low income participating in FVI programs (*n* = 12) (13); when combining 7 of the 12 like studies, Stein et al. reported an ~18 percentage point reduction in the percentage of individuals with food insecurity. A recent randomized control study evaluated a healthy food benefit program for households with low income in Seattle (11). Knox et al. (11) report that a $40 per month benefit to buy fruits and vegetables at a wide range of retailers was associated with a decrease in food insecurity. Likewise, a study of >20,000 adult SNAP participants suggested that >6 months of participation (versus first-time participation) in the Gus Schumacher Nutrition Incentive (GusNIP) was associated with ~40% lower odds of having food insecurity (12). However, several key methodological differences between prior literature and our study warrant consideration. Most importantly, most studies included in the review (13) and the large GusNIP study by Shanks et al. (12) did not include control groups. Moreover, for many studies reporting on food insecurity, program duration was <6 months or not reported and most FVI were provided via a subsidy (i.e., a fixed amount of money) versus a match (i.e., money tied to dollars spent) (13). Additionally, the study conducted by Knox et al. (11) and the review (13) did not include studies that distributed FVI through larger federal nutrition assistance programs; although 55% of individuals included in the systematic review (13) were participating in SNAP. Although none of these prior studies examined nutrition security, our null findings also diverge from literature suggesting FVI programs improve FV intake (11, 13)—a proxy likely correlated with nutrition security.

There are other plausible explanations for our null findings. In our sample, only ~30% of individuals were nutrition insecure, which is lower than the prevalence of nutrition insecurity (~50%) reported by Tucker et al. (5) and Calloway et al. (6) The lower prevalence in our sample may be because a large proportion also received WIC benefits, which likely provides an additional buffer against both food and nutrition insecurity. However, it is relevant to note the decrease in WIC participation in the follow-up period. Additionally, it is possible that our follow-up period was too short (5–8 months) to observe significant changes in our outcomes. Further research, with a longer follow-up period, is important to help evaluate the longer-term impact and economic feasibility of sustained FVI programs. It is also possible that the incentive size, restricting to fresh FV, limited program awareness, and implementation occurring only at two store chains were limiting factors. Although our null results were unexpected, they are generally consistent with our primary analyses, which found a non-significant but positive association between FVI and fruit and vegetable intake overall (14). Our null findings may also be a result of limited program awareness as only one-quarter of the RI sample reported using the EWBW incentives after the policy was implemented (14), which underscores the need for effective communication strategies to accompany financial incentives, as has been demonstrated in other nutrition policy evaluations (26).

This study is subject to several limitations. First, nutrition security was assessed using a 1-item screener that has not yet been used extensively in the literature; however, the 1-item measure was well-correlated with the 4-item validated measure at follow-up (adjusted OR: 5.95; 95% CI: 4.40–8.04; data not shown in tables). Nevertheless, the development of a more robust nutrition security measure using a rigorous, mixed-methods approach like that employed to develop the 18-item USDA Household Food Security Survey Module warrants consideration (27). Moreover, both food and nutrition security are self-reported, which could introduce recall or social desirability bias. Our sample included predominantly women, most participants were also receiving WIC benefits (~70%), and our evaluation is of one statewide program in the Northeast, which may limit our external generalizability. Additionally, participant attrition over time could limit the external generalizability of results. Although CT was chosen as the comparison state based on demographic, socioeconomic, and political similarity, we cannot rule out the possibility of unmeasured confounding stemming from policy or contextual differences between RI and CT that changed over the course of the study. This study also has several strengths including our large sample of SNAP participants, evaluation of a statewide automatic FVI, quasi-experimental design, and inclusion of a nutrition security measure.

## Conclusion

The EWBW FVI was not associated with food or nutrition insecurity among SNAP participants ~6 months after implementation. The results of this short-term follow-up suggest that providing an automatic nutrition incentive to EBT cards for use at a limited number of retailers may be insufficient to reduce food and nutrition insecurity. Future analyses should evaluate the effects of the FVI program after a longer follow-up duration and how factors like incentive size, store selection, and restrictions on incentive use work in concert to affect food and nutrition insecurity.

## Data Availability

The raw data supporting the conclusions of this article will be made available by the authors, without undue reservation.
